# The relative impact of vision impairment and cardiovascular disease on quality of life: the example of pseudoxanthoma elasticum

**DOI:** 10.1186/1477-7525-9-113

**Published:** 2011-12-12

**Authors:** Robert P Finger, Eva Fenwick, Manjula Marella, Peter Charbel Issa, Hendrik PN Scholl, Frank G Holz, Ecosse L Lamoureux

**Affiliations:** 1 Department of Ophthalmology, University of Bonn, Ernst-Abbe-Strasse 2. D-53127,Bonn, Germany; 2 Centre for Eye Research Australia, Royal Victorian Eye and Ear Hospital, Peter Howson WingLevel 1, 32 Gisborne Street, East Melbourne VIC, 3002 Australia, University of Melbourne, Melbourne, Australia; 3 Nuffield Laboratory of Ophthalmology, University of Oxford, Level 5 and 6, West Wing, The John Radcliffe Hospital, Headley Way, OX3 9DU, Oxford, UK; 4 Wilmer Eye Institute, Johns Hopkins University School of Medicine, Baltimore, Maryland, USA; 5 Singapore Eye Research Institute, Singapore National Eye Centre, Singapore

**Keywords:** Vision-related quality of life (VRQoL), health-related quality of life (HRQoL), visual impairment, cardiovascular disease, Pseudoxanthoma elasticum (PXE), Impact of Vision Impairment Questionnaire (IVI), SF-36

## Abstract

**Objective:**

To investigate the impact of pseudoxanthoma elasticum (PXE), a rare hereditary disease of concurrent vision impairment (VI) and cardiovascular complications (CVCs), on vision-related (VRQoL) and health-related quality of life (HRQoL).

**Methods:**

VRQoL and HRQoL were assessed using the Impact of Vision Impairment (IVI) questionnaire and the Short Form Health Survey (SF-36) in 107 PXE patients. Patients were stratified into four groups: A = no VI or CVC; B = CVCs only; C = VI only; and D = both VI and CVCs.

**Results:**

Following Rasch analysis, the IVI was found to function as a vision-specific functioning and emotional well-being subscale, and the SF-36 as a health-related physical functioning and mental health subscale. The presence of VI and CVC were significant predictors of vision-specific functioning and emotional well-being (p < 0.001), with a clinically meaningful decrement in vision-specific functioning in patients with VI. No associations were found for the SF-36 Physical Functioning and Mental Health scores between any groups.

**Conclusions:**

Vision impaired patients with PXE report significantly poorer vision-specific functioning than PXE patients without VI. In contrast, the relative impact of PXE on reported general HRQoL was much less. Our results suggest that vision impairment has the larger impact on QoL in this sample.

## Introduction

Pseudoxanthoma elasticum (PXE) is a rare, hereditary, autosomal recessive disease [1]. PXE is characterized by a systemic calcification of elastic tissue affecting foremost the skin, the ocular fundus and the cardiovascular system. Cardiovascular manifestations of PXE include arterial hypertension, peripheral arterial disease, angina pectoris, restrictive cardiomyopathy, mitral valve prolapse or stenosis, and sudden cardiac failure, often resulting in death [2-7]. PXE also affects the ocular fundus due to a centrifugal alteration of Bruch's membrane [1,8]. This eventually leads to breaks in Bruch's membrane which may appear clinically as angioid streaks [9], predisposing the patient to the development of choroidal neovascularisations (CNVs). These secondary angiogenic processes usually occur as early as the third or fourth decade of life, leading to the vast majority of patients being legally blind in their fifth or sixth decade [1].

Vision impairment (VI) and cardiovascular complications (CVCs) have been shown to adversely affect daily functioning and other aspects of quality of life (QoL) [10-14]. Consequently, it can be hypothesised that PXE patients, who have both VI and CVCs, will experience poor vision-related (VRQoL) and health-related quality of life (HRQoL). However, to date no attempt has been made to quantify the VRQoL or HRQoL impact of PXE from the patient's perspective. Similarly, it remains unknown whether the magnitude of the impact of VI and CVCs on VRQoL or HRQoL is similar, or whether one is more detrimental than the other. This information is essential for rehabilitation workers and policy planners to develop optimal services and resources.

Therefore, we investigated the magnitude of the impact of PXE on VRQoL and HRQoL using the Impact of Vision Impairment questionnaire (IVI)[15,16] and the Short Form Health Survey (SF-36)[17,18], respectively, in a sample of PXE patients with differing levels of VI and CVCs.

## Methods

### Patients

A total of 198 German patients with PXE were sent a postal survey in 2008 using the mailing list of the German PXE Patient Association, of whom 135 returned completed questionnaires (response rate 68%). Each participant received the IVI and SF-36 questionnaires; a short questionnaire assessing the patients' sociodemographic characteristics and medical history; and a consent form. Self-reported medical history, including ophthalmic history, was validated against available responding patients' files known to the department of ophthalmology at the University of Bonn (n = 82). Based on very limited data available, respondents and non-respondents seemed no different. However, too limited data was available for non-responders to allow for a statistical comparison. Ethical approval was obtained from the ethics committee of the University of Bonn. All patients consented to partaking in the study. The study adhered to the tenets of the declaration of Helsinki.

### Quality of life outcome measures

#### Impact of Vision Impairment (IVI)

The IVI questionnaire is a vision-specific instrument which measures the impact of vision impairment on various QoL parameters and was developed using focus group discussions and input from existing instruments [19]. The IVI contains 28 items with 4-5 response options using Likert scaling, ranging from '*not at all' *to '*can't do because of eye sight'*. Items form three specific subscales: 'reading and accessing information', 'mobility and independence' and 'emotional well-being'. The IVI has been shown to be reliable, [20] responsive to interventions [16] and it has been rigorously validated using modern psychometric methods such as Rasch analysis for different ocular conditions as well as levels of visual impairment [15,16,21]. The psychometric properties of the German IVI have recently been evaluated by our group using Rasch analysis and it was found to be a valid and reliable outcome measure to assess VRQoL[22].

#### Short Form Health Survey (SF-36)

The SF-36 is a generic health-related QoL tool which has been validated across a number of populations with various conditions, both chronic and acute [17,23-25]. The SF-36 measures eight dimensions of health and well-being using 36 items which are coded, summated and transformed to yield eight subscales. These can be further reduced into two domains, namely the physical and mental component score. The German version has been thoroughly validated and used to collect normative data across a broad spectrum of health states, including healthy controls [18,26,27].

### Psychometric Validation of the IVI and the SF-36

Rasch analysis is a modern psychometric technique that calculates person ability in relation to item difficulty by placing them on the same linear continuum. Rasch analysis provides insight into the psychometric properties of a scale, such as its reliability and overall fit to the model, the appropriateness of the response scale used, unidimensionality, targeting of the scale to the sample involved, and individual item fit and item bias. In Rasch analysis, raw ordinal scores are transformed into estimates of interval-level measurement (expressed in log of the odds units, or logits). A high logit score indicates that a person possesses a high level of the assessed latent trait (e.g. VRQoL). To ease interpretation, the rating scale of the IVI was reversed for Rasch analysis so that patients with a high level of VRQoL were given high scores. The rating scale for the SF-36 items was not reversed as the most able participants were already allocated the highest score.

Rasch analysis was undertaken using the Andrich rating scale model [28] with Winsteps software (version 3.68), Chicago, Illinois, USA [29] to validate both the IVI and the SF-36. Several key indicators of each scale were examined. We assessed the response category threshold ordering by visually checking for disordered thresholds. Disordered thresholds may result when a category is underused, category definition is unclear, or when participants have difficulty discriminating between response options. Disordered thresholds can cause significant item and model misfit and collapsing response categories may be necessary to improve model fit. The discriminant ability of the scale was determined using the person separation index (PSI) and person reliability (PR) values which measure the ability of the scale to distinguish distinct levels of participant ability. A PSI of 2.0 and a person reliability score of 0.8 represent three distinct levels of participant ability [30]. Targeting of item difficulty to participant ability is assessed by inspecting the person-item map, where the person and item measures are displayed on the same calibration ruler. Effective targeting is evident when the person and item means (in logits) are similar. By default, the mean item value is zero [31].

Rasch analysis requires that a scale measures a single underlying trait, or that it is unidimensional. Thus we tested all conventional subscales for the IVI and SF-36 as well as summary scores. Two parameters are used to assess scale unidimensionality: item 'fit statistics' and testing the assumption of local independence. Item fit determines how well each item fits the underlying trait, e.g., VRQoL and items with an infit mean square value (MNSQ) ranging between 0.7 and 1.3 were considered acceptable. The primary component analysis (PCA) of the residuals was examined to test for local independence. The variance explained by the Rasch measures for the empirical calculation should be comparable to that of the model (> 50% for an acceptable model). Furthermore, the unexplained variance by the residuals in the first contrast should be < 2.4 eigenvalue units which is close to that seen with random data. Finally, we assessed for differential item functioning (DIF) which indicates whether different groups within the sample (e.g. gender, age) systematically respond differently despite equal levels of the trait being assessed. A DIF contrast of > 1.0 logits for an item was considered to represent notable DIF and to indicate possible interpretation bias for that item.

### Statistical Analysis

The SPSS statistical software (Version 17.0, SPSS Science, Chicago, IL) was used to analyze the data. Patients were stratified into four groups according to their clinical characteristics, namely Group A = no VI and no CVC (n = 16); Group B = CVCs only (n = 35); Group C = VI only (n = 15); and Group D = both VI and CVCs (n = 41). Descriptive statistical analyses were performed to characterize the participants' sociodemographic, clinical, IVI and SF-36 data using univariate analyses of variance for continuous variables and multinomial logistic regression for categorical variables. Functional and emotional domain scores of both HRQoL and VRQoL were the main outcomes. Following Rasch analyses, the overall and individual person scores were obtained as linear estimates, which then were fitted to regression models. The association between VRQoL and HRQoL (overall and specific aspects of) and PXE was analysed using regression models, adjusting for covariables that were found to be univariately associated with the main outcomes i.e. age, gender and visual impairment. Partial eta-squared which is a measure of effect size was used to describe the strength of the association between a predictor (or set of predictors) and the dependent variable. It can be characterized as the proportion of total variation attributable to the factor, partialling out (excluding) other factors from the total nonerror variation [32].

Twenty-eight patients were removed from the final analyses as they were without vision or general impairment data or too able for the questionnaires as evident by a ceiling effect in their item responses. As with all questionnaires, those participants experiencing little disability from the assessed health condition may find the questions very easy - in other words the questions are too easy for very able participants. This can affect the targeting of the questionnaire, meaning that the mean difficulty of the items does not effectively match the mean ability of the participants. Thus, by removing the most able participants from the analysis the targeting, and consequently the overall functioning of the scale, improves. This resulted in a final sample size of 107 participants. Sample size required by Rasch analysis is calculated by items per questionnaire times 5 responders for a validation study. As both questionnaires have been previously validated in German, the slightly smaller sample size than no. of items × 5 in this study can still be considered sufficient, in particular considering the rarity of PXE [33].

## Results

### Sample Characteristics

The majority of the sample was female (n = 68, 63%; Table 1). The mean ± SD age and best corrected visual acuity values were 57 ± 12 years and 0.79 ± 0.67 LogMAR, respectively. Patients had, on average, 1.6 CVCs with hypertension, peripheral arterial disease and coronary heart disease being most common. Over forty percent of the patients (n = 45, 42%) needed help filling out the questionnaires. After splitting the sample into four groups, patients with VI and CVCs (group D) were significantly older than the other groups (all p≤0.05) and patients with VI (groups C & D) needed more help to fill in their questionnaires (all p≤0.01). The unequal gender distribution (63% women) was similar across all four subgroups (p > 0. 05).

**Table 1:** Sociodemographic and clinical characteristics of the 107 patients with PXE.

	Total sample (n = 107)	Group A (n = 16) No VI or CVCs	Group B (n = 35) Only CVCs	Group C (n = 15) Only VI	Group D (n = 41) VI & CVCs	p value*
**n (%)**						
Gender						0.917
Male	38 (36%)	5 (31%)	14 (40%)	4 (27%)	15 (37%)	
Female	68 (64%)	11 (69%)	21 (60%)	10 (67%)	26 (63%)	
Help to fill in questionnaires (self-report)	45 (42%)	1 (6%)	3 (9%)	9 (60%)	32 (78%)	< 0.001
**Mean ± SD**						
Age (years)	57 ± 12	47 ± 11	53 ± 10	54 ± 16	64 ± 9	< 0.001
BCVA Snellen (LogMAR)	20/125 (0.79 ± 0.67)	20/25 (0.11 ± 0.11)	20/32 (0.16 ± 0.16)	20/250 (1.10 ± .056)	20/320 (1.24 ± 0.53)	< 0.001
Average no. of CVCs	1.61 ± 1.39	0	2.09 ± 1.12	0	2.41 ± 1.07	< 0.001
Duration of PXE (years)	17 ± 12	11 ± 10	18 ± 13	20 ± 14	18 ± 9	0.127
Functional IVI score	20.16 ± 8.16	28.12 ± 4.99	23.52 ± 6.62	20.99 ± 6.18	13.63 ± 5.99	< 0.001
Emotional IVI score	8.82 ± 3.24	10.75 ± 2.46	9.06 ± 3.11	10.63 ± 3.05	7.22 ± 2.98	< 0.001
Rasch guided SF-36 physical functioning score	19.98 ± 7.58	23.21 ± 5.73	19.76 ± 8.02	16.93 ± 7.35	20.29 ± 7.58	0.141
Rasch guided SF-36 mental health score	14.48 ± 6.19	16.77 ± 6.04	13.91 ± 5.67	14.30 ± 5.93	14.35 ± 6.69	0.478

SD = Standard deviation; CVCs = cardiovascular complications; VI = Vision impairment; BCVA = Best corrected distance visual acuity. *ANOVA, testing for difference between all groups.

### Psychometric evaluation of the German IVI and SF-36

#### Psychometric properties of the IVI

The data for the German-translated IVI were fitted to the Rasch model and several indicators of fit were explored (Table 2). There was evidence of disordered thresholds which necessitated categories 1 and 2 ('*a fair amount' *and '*a little'*) to be collapsed, resulting in ordered thresholds for all items. The PSI and the PR values were 4.07 and 0.94, respectively, which indicates that the scale was able to discriminate between five strata of VRQoL. The targeting of the instrument was acceptable (difference in person and item means 1.15 logits). However, there was evidence of multidimensionality in the scale. Although the raw variance explained by the PCA of the residuals was adequate (64.2%), the unexplained variance in the first contrast of the residuals was 3.9, suggesting the existence of a second dimension. Moreover, four items (Items 21, 22, 25, 26) demonstrated misfit (MNSQ > 1.3). All four of these items belonged to the 'emotional well-being' domain and their standardized residual loadings were all > 0.4 units suggesting that they were loading onto the same construct. Removal of these items did not improve the overall fit statistics. Therefore, the IVI was split into a Functional Scale (Items 1-20) and Emotional Scale (Items 21-28) which resulted in both scales fitting the Rasch model (Table 3).

**Table 2:** Fit parameters of the IVI (and subscales) and SF-36 scales compared to Rasch model requirements.

Parameters	Rasch model	IVI_O	IVI_F	IVI_E	SF36_C	SF-36_PF	SF-36_MH
Disordered thresholds	No	**Yes**	No	No	No	No	No
No. of misfitting items	0	**4**	0	**1**	**4**	0	**1**
Person Separation Index	> 0.2	4.07	4.28	2.42	2.66	2.64	2.13
Person Reliability	> 0.8	0.94	0.95	0.85	0.88	0.87	0.82
Person-item mean difference	< 1	**1.15**	**1.25**	**1.49**	0.30	**2.25**	**1.26**
Variance by 1^st ^factor	> 50%	64.2%	63.3%	63.1%	**40.7%**	68.2%	66.5%
PCA (Eigenvalue for 1^st ^contrast)	< 2.4	**3.9**	**2.4**	1.8	**8.3**	**2.3**	**2.2**
Differential Item Functioning							
Gender	< 1.0	**NA**	**None**	**None**	**NA**	**None**	**None**
Age group (≤50, > 50)	< 1.0	**NA**	**None**	**None**	**NA**	**None**	**None**
Vision impairment (VI, non-VI)	< 1.0	**NA**	**None**	**None**	**NA**	**None**	**None**
Participant person measure mean ± SD	-	**NA**	20.16 ± 8.16	8.82 ± 3.24	**NA**	19.98 ± 7.58	14.48 ± 6.19
Clinically meaningful cut-off	-	**NA**	< -4.1, > 4.1	< -1.6, > 1.6	**NA**	< -3.8, > 3.8	< -3.1, > 3.1

IVI_O = Impact of Vision Impairment Original; IVI_F = Impact of Vision Impairment Functional; IVI_E = Impact of Vision Impairment Emotional; PCA = Principle Components Analysis; SD = Standard deviation; SF36_C = SF36 Complete; PF = Physical Functioning scale of the SF-36, MH = Mental Health scale of the SF-36; NA = Not assessed; VI = Vision Impairment

Bolded cells indicate misfiting values compared to the Rasch model requirements

**Table 3:** Model characteristics and differences between the four groups, after adjusting for age, gender, VI, number of comorbidities other than CVCs, duration of PXE and help needed to fill in questionnaires (analysis of covariance).

	Adjusted mean/RC*	95% CI	Significance of the model/change*	Partial Eta^2 +^	
				Corrected model	Groups A-D as a predictor within the model
**Functional IVI**			**p < 0.001**	**0.812^+^**	**0.241^+^**
Group A (reference)	23.66	(20.92; 26.40)			
Group B	-1.89	(-4.91; 1.13)	0.216		
Group C	-5.60	(-9.19; -2.01)	**0.003**		
Group D	-6.90	(-10.41; -3.39)	**< 0.001**		
**Emotional IVI**			**p < 0.001**	**0.662^+^**	**0.128^+^**
Group A (reference)	7.47	(5.97; 8.97)			
Group B	-0.33	(-1.88; 1.22)	0.777		
Group C	1.94	(0.06; 3.82)	**0.043**		
Group D	1.32	(-0.66; 3.31)	0.188		
**Rasch guided SF-36 Physical Functioning**			p = 0.086	0.187^+^	0.064^+^
Group A (reference)	21.50	(15.51; 27.49)			
Group B	-2.34	(-8.66; 3.97)	0.461		
Group C	-6.03	(-13.49; 1.45)	0.111		
Group D	-0.81	(-8.70; 7.09)	0.839		
**Rasch guided SF-36 Mental Health**			p = 0.093	0.159^+^	0.044^+^
Group A (reference)	16.28	(12.06; 20.50)			
Group B	-3.77	(-8.41; 0.87)	0.109		
Group C	-0.19	(-5.87; 5.49)	0.947		
Group D	-2.28	(-7.64; 3.09)	0.400		

Presented are fully adjusted models including interactions between all variables.

Functional and emotional subscale scores of both the IVI and SF-36 contributed reciprocally and were also adjusted for.

RC = regression coefficient, *reported for the model/change of mean in groups compared to group A (reference); CI = Confidence interval; Group A = No impairment; Group B = Only CVCs; Group C = Only VI; Group D = CVCs & VI; **Bolded **values indicate statistical significance; **^+ ^**Partial Eta^2 ^effect size: ≥0.01 small, ≥0.06 medium, ≥0.14 large effect[53]

The Functioning Scale had excellent discriminant ability, no misfitting items, and minimal evidence of multidimensionality with the PCA for the first factor explaining > 60% of the variance and the first contrast of the residuals being acceptable (2.4 eigenvalues). Targeting was suboptimal (difference in person and item mean 1.25) which may suggest that the patients in this sample had a higher level of ability than the average difficulty of the IVI items. No DIF was found for age group, gender or VI.

The Emotional Scale had adequate discriminant ability and satisfied the requirements for unidimensionality. One item (Item 21) displayed misfit (MNSQ 1.64 logits), however, it was retained as deleting it did not improve fit statistics and it captures important emotional information, i.e. embarrassment caused by eyesight. No DIF was found for age group, gender or VI. Again, the targeting of this subscale suggested that patients in this sample were of higher ability than the average item difficulty of the IVI.

#### Psychometric properties of the SF-36

First, all eight conventional SF-36 subscales as well as the conventional summary scores were tested using Rasch analysis, but none met the requirements of the Rasch model. Thus we continued with Rasch analysis of the overall item pool to arrive at Rasch guided subscales. The overall SF-36 scale had no disordered thresholds indicating that the number and clarity of response options were appropriate. The PSI and PR values were 2.66 and 0.88, respectively, which indicates satisfactory discriminant ability of the scale. Targeting of the scale was also excellent (difference in person and item mean 0.30 logits). For the overall SF-36, there were four misfitting items (Items 20, 21, 22, 35) and evidence of multidimensionality (PCA of the residuals < 50% and unexplained variance in the first contrast of the residuals 8.3). Deletion of misfitting items did not improve any of the fit statistics. The standardized residual loadings of the SF-36 items were explored to assess whether items were loading onto separate factors. Items pertaining to functional and emotional well-being loaded as separate subscales. Therefore, we fitted the physical component domain (items 3a-3j, 4a-d, 7-8, 11a-d) and mental component domain (items 5a-c, 6, 9a-h, 10, 11a-d) of the SF-36 to the Rasch model. After assessing all model fit statistics, the SF-36 was eventually split into a 10-item Physical Functioning scale (items 3a-3j) and a 5-item Mental Health scale (items 9b, c, d, f, h).

The Physical Functioning scale displayed satisfactory discriminant ability and unidimensionality. However, the targeting of this scale was not optimal with a mean difference between person and items of 2.25, suggesting that this sample was much more able than the average item difficulty of the scale. The Mental Health scale also demonstrated adequate discriminant ability and unidimensionality. One item displayed borderline misfit (Item 9h, MNSQ 1.34); however, since removal of this item did not improve other fit statistics it was retained. No DIF was found for gender, age group or VI for either the physical functioning or mental health scales. These results collectively show that the Functional and Emotional IVI and Physical Functioning and Mental Health SF-36 subscales are unidimensional, reliable and valid scales to assess VRQoL and HRQoL, respectively, in this population.

To facilitate the interpretation of the person measure scores, they were recalibrated from a negative-positive scale to range between 0 and 40 for the IVI functional subscale, 0 and 16 for the IVI emotional subscale, 10 and 30 for the SF-36 physical functioning subscale and 5 and 30 for the SF-36 mental health subscale. These values represent the minimum and maximum possible summed values for each subscale. In linear regression models, independent significant predictors of VRQoL and HRQoL were considered to be clinically meaningful if the confidence interval limits of their beta coefficients were approximately half the standard deviation of the overall mean. This is generally considered to be a useful estimate of a clinically meaningful difference [34,35]. The participants' mean ± SD score and clinically meaningful cut-offs for each of the four final scales are given in Table 3.

### Relationship between vision impairment, cardiovascular complications and VRQoL/HRQoL

Functional and emotional IVI scores were lowest in patients with VI and CVC (Group D, p = 0.001 and 0.049, respectively) compared to all other groups (Table 1). In adjusted regression models, the presence of VI and CVC were significant predictors of visual functioning and emotional well-being compared to group A (no VI and CVC) (partial Eta^2 ^0.812 and 0.662, respectively; p < 0.001; Table 3). No association was however found for the SF-36 Physical Functioning and Mental Health scale scores (Table 3).

After controlling for age, gender, duration of PXE, number of comorbidities other than CVCs, help needed to fill in the questionnaires (as a surrogate measure of disability) and self-reported general health; vision impaired patients of groups C (-5.6; p = 0.003 and 0.019 compared to groups A & B, respectively) and D (-6.9; p < 0.001 compared to groups A & B) reported poorer vision-specific functioning than groups A (reference) and B (-1.89) who did not have any VI. These results also represent a clinically meaningful reduction of vision-related functioning in vision-impaired PXE patients based on our estimated cut-off values. No significant difference was found for vision-specific functioning and emotional well-being between groups C and D (p = 0.388 and 0.413, respectively), despite the presence of CVCs in group D. Group B (-0.33) reported poorer vision-specific emotional well-being compared to groups C (1.94, p = 0.005) and D (1.32, p = 0.029, group-wise comparison not shown in Table 3) despite not being vision impaired. Vision-specific emotional well-being did not differ between groups A and D (p > 0.05) and was slightly higher in group C compared to A (p = 0.043). The SF-36 Physical Functioning and Mental Health scores were not significantly different between any of the groups (p > 0.05).

## Discussion

We investigated the impact of PXE on VRQoL and HRQoL in this sample. Our findings indicate a considerable and clinically meaningful impact on visual functioning and a moderate association with vision-specific emotional well-being in PXE patients with VI, irrespective of the presence of CVCs. The impact on HRQoL was found to be minimal despite considerable concurrent cardiovascular disease in a large proportion of the sample, indicating a larger impact of vision impairment than cardiovascular disease on reported QoL in this sample.

The detrimental impact of VI on visual functioning has been shown for a number of ocular conditions[21,36,37] and systemic diseases with ocular complications[38,39] which is consistent with the main finding from our study. Vision rehabilitation has been shown to improve participation in activities of daily living in both patients with primary ocular disease [40] and patients with ocular complications from systemic diseases who may sometimes also have severe additional impairments [39,41,42]. Therefore, timely referral of patients for visual rehabilitation is crucial for maintaining good visual functioning and QoL.

The finding that PXE patients without VI had worse vision-specific emotional well-being than those with VI is seemingly contradictory. It may be due to the anxiety experienced by non visually impaired PXE patients predicting the almost certain occurrence of ocular complications and the possibility of future bilateral blindness, despite available treatment [1,43]. In patients with age-related macular degeneration, the prospect of losing vision in the first eye has been shown to lead to much higher levels of anxiety and stress in newly affected patients compared to a further loss of vision in the remaining eye in patients at the more severe spectrum of disease [37]. Similarly, studies in patients with diabetic retinopathy have found that those experiencing recent disease progression and fluctuating vision report more negative life events[44] and higher levels of psychological distress, especially depression, than those with worse yet stable vision [45]. These phenomena may reflect differing levels of personal experience, better coping strategies and adaptation to disease progression and level of VI in patients with long-standing VI [37]. In addition, the unusual finding of better vision-specific emotional well-being in patients with VI compared to patients without VI may be partly due to patients with VI having access to a highly specialized service including personalized follow-up at the department of ophthalmology at the University of Bonn. Comprehensive low vision rehabilitation programmes have repeatedly been shown to improve not only functional ability but also emotional well-being in visually impaired patients [46-48]. Alternatively, this may in fact be a spurious outcome due to our small sample size following stratification. Therefore, future studies are needed to confirm this finding.

No similarly specialised services within a clinic setting dedicated to PXE are available to PXE patients with CVCs in Germany. Access to such services has been shown to improve the emotional well-being of patients suffering from similar physical impairments. For example, in a study of HRQoL in Ehlers-Danlos syndrome, another rare disease of the connective tissue, patients' emotional well-being as assessed by the SF-36, was shown to be positively influenced by access to a highly specialized service unit [49].

Given the rarity of PXE, having such a large sample of patients with varying degrees of VI and general impairment caused by cardiovascular disease, is a major strength of this study. Further strengths include the use of Rasch analysis, an important step in modern scale validation, to assess the psychometric properties of the German IVI and SF-36, and to produce estimates of interval-level measurements of vision-specific functioning and emotional well-being, and general health-related physical functioning and mental health. Use of both scales provides a comprehensive assessment of the impact of low vision and cardiovascular disease on vision-specific and health-related QoL parameters. The use of two QoL scales to assess the impact of multiple impairments in this study is justified as only minimal correlation has been found between VRQoL and HRQoL scores [50,51]. Indeed, the results of this study convincingly demonstrate the importance of using a vision-specific outcome measure rather than a generic HRQoL outcome measure to assess the impact of VI on VRQoL. Generic patient-reported outcomes (PROs) have very little vision-related content[52] and it is unlikely that any impact of VI on generic health-related QoL will be successfully captured by these instruments.

Conversely, our study is limited by a sample size which in general terms has to be considered small which includes a high number of patients with no significant impairment and future studies would benefit from a greater proportion of patients with low vision and/or general disability. Use of Rasch analysis is appropriate for smaller sample sizes as the response patterns on which the analysis is based are not as easily skewed as raw scores generated by classical test theory. Furthermore, selection of our sample may be biased due to a response rate of 68%. However, respondents and non-respondents did not seem to differ, and given the rarity of PXE, larger studies are very difficult to conduct. The use of the SF-36, a generic HRQoL measure rather than a CVC specific measure, was felt justified as PXE is a connective tissue disease leading to a broad range of complications such as disfiguring skin changes, mobility restriction, pain and gastrointestinal bleeding. We felt that the impact of such a range of ailments could potentially be better captured by a general HRQoL instrument than a CVC-specific PRO measure. However, future studies that include measures of disability caused by cardiovascular disease such as actual walking distance or pain, as well as measures of anxiety and/or depression may add to the understanding of the impact of PXE on HRQoL.

## Conclusion

In conclusion, our novel study assessing the impact of PXE on VRQoL and HRQoL demonstrates that there is a significant impact on vision-specific functioning and emotional well-being in PXE patients with and without VI, irrespective of the presence of other CVCs. In contrast, little impact on general HRQoL was found. Therefore, these results indicate that PXE patients would benefit from specialized service provision focusing on visual rehabilitation. Based on these findings, vision impairment seems to have a larger impact than cardiovascular disease on reported QoL in PXE patients.

## Competing interests

The authors declare that they have no competing interests.

## Authors' contributions

ARPF, PCI, HPNS, FGH have made substantial contributions to conception and design, acquisition of data, RPF, EF, ELL and PCI to analysis and interpretation of data; RPF, MM, PCI, HPNS, FGH, EF, ELL have been involved in drafting the manuscript or revising it critically for important intellectual content; and all authors have given final approval of the version to be published.

## References

[B1] Finger RP Charbel Issa P Ladewig MS Gotting C Szliska C Scholl HP Holz FG Pseudoxanthoma elasticum: genetics, clinical manifestations and therapeutic approaches Surv Ophthalmol 2009 54 272 285 10.1016/j.survophthal.2008.12.006 19298904

[B2] Challenor VF Conway N Monro JL The surgical treatment of restrictive cardiomyopathy in pseudoxanthoma elasticum Br Heart J 1988 59 266 269 10.1136/hrt.59.2.266 3342167 PMC1276996

[B3] Fukuda K Uno K Fujii T Mukai M Handa S Mitral stenosis in pseudoxanthoma elasticum Chest 1992 101 1706 1707 10.1378/chest.101.6.1706 1600795

[B4] Lebwohl M Halperin J Phelps RG Brief report: occult pseudoxanthoma elasticum in patients with premature cardiovascular disease N Engl J Med 1993 329 1237 1239 10.1056/NEJM199310213291705 8413390

[B5] Lebwohl MG Distefano D Prioleau PG Uram M Yannuzzi LA Fleischmajer R Pseudoxanthoma elasticum and mitral-valve prolapse N Engl J Med 1982 307 228 231 10.1056/NEJM198207223070406 7088072

[B6] Scheie HG Hogan TF Jr Angioid streaks and generalized arterial disease AMA Arch Ophthalmol 1957 57 855 868 10.1001/archopht.1957.00930050869010 13423951

[B7] Cailleux N Hachulla E Perez-Cousin M Labalette P Lecomte-Houcke M Pseudoxanthoma elasticum: a rare cause of leg artery diseases in young adults J Mal Vasc 1997 22 51 55 9120374

[B8] Charbel Issa P Finger RP Gotting C Hendig D Holz FG Scholl HP Centrifugal fundus abnormalities in pseudoxanthoma elasticum Ophthalmology 2010 117 1406 1414 10.1016/j.ophtha.2009.11.008 20189652

[B9] Charbel Issa P Finger RP Holz FG Scholl HP Multimodal imaging including spectral domain OCT and confocal near infrared reflectance for characterisation of outer retinal pathology in pseudoxanthoma elasticum Invest Ophthalmol Vis Sci 2009 10.1167/iovs.09-3541 19553619

[B10] West SK Munoz B Rubin GS Schein OD Bandeen-Roche K Zeger S German S Fried LP Function and visual impairment in a population-based study of older adults. The SEE project. Salisbury Eye Evaluation Invest Ophthalmol Vis Sci 1997 38 72 82 9008632

[B11] Mangione CM Gutierrez PR Lowe G Orav EJ Seddon JM Influence of age-related maculopathy on visual functioning and health-related quality of life Am J Ophthalmol 1999 128 45 53 10.1016/S0002-9394(99)00169-5 10482093

[B12] Bach JP Riedel O Pieper L Klotsche J Dodel R Wittchen HU Health-Related Quality of Life in Patients with a History of Myocardial Infarction and Stroke Cerebrovasc Dis 31 68 76 10.1159/000319027 21051886

[B13] Borowiak E Kostka T Influence of chronic cardiovascular disease and hospitalisation due to this disease on quality of life of community-dwelling elderly Qual Life Res 2006 15 1281 1289 10.1007/s11136-006-0058-0 16977423

[B14] Sprengers RW Teraa M Moll FL de Wit GA van der Graaf Y Verhaar MC Quality of life in patients with no-option critical limb ischemia underlines the need for new effective treatment J Vasc Surg 52 843 849 849 e841 10.1016/j.jvs.2010.04.057 20598482

[B15] Lamoureux EL Pallant JF Pesudovs K Hassell JB Keeffe JE The Impact of Vision Impairment Questionnaire: an evaluation of its measurement properties using Rasch analysis Invest Ophthalmol Vis Sci 2006 47 4732 4741 10.1167/iovs.06-0220 17065481

[B16] Lamoureux EL Pallant JF Pesudovs K Rees G Hassell JB Keeffe JE The impact of vision impairment questionnaire: an assessment of its domain structure using confirmatory factor analysis and rasch analysis Invest Ophthalmol Vis Sci 2007 48 1001 1006 10.1167/iovs.06-0361 17325138

[B17] Ware JE Jr Sherbourne CD The MOS 36-item short-form health survey (SF-36). I. Conceptual framework and item selection Med Care 1992 30 473 483 10.1097/00005650-199206000-00002 1593914

[B18] Bullinger M German translation and psychometric testing of the SF-36 Health Survey: preliminary results from the IQOLA Project. International Quality of Life Assessment Soc Sci Med 1995 41 1359 1366 10.1016/0277-9536(95)00115-N 8560303

[B19] Keeffe JE McCarty CA Hassell JB Gilbert AG Description and measurement of handicap caused by vision impairment Aust N Z J Ophthalmol 1999 27 184 186 10.1046/j.1440-1606.1999.00179.x 10484186

[B20] Weih LM Hassell JB Keeffe J Assessment of the impact of vision impairment Invest Ophthalmol Vis Sci 2002 43 927 935 11923230

[B21] Lamoureux EL Hassell JB Keeffe JE The determinants of participation in activities of daily living in people with impaired vision Am J Ophthalmol 2004 137 265 270 10.1016/j.ajo.2003.08.003 14962415

[B22] Finger R Fenwick E Marella M Petrak M Holz F Chiang P Lamoureux E The impact of vision impairment on vision-specific quality of life in Germanyunder review 2011 10.1167/iovs.10-7127 21357395

[B23] Ware JE Jr Kosinski M Gandek B Aaronson NK Apolone G Bech P Brazier J Bullinger M Kaasa S Leplege A The factor structure of the SF-36 Health Survey in 10 countries: results from the IQOLA Project. International Quality of Life Assessment J Clin Epidemiol 1998 51 1159 1165 10.1016/S0895-4356(98)00107-3 9817133

[B24] Gandek B Ware JE Jr Aaronson NK Alonso J Apolone G Bjorner J Brazier J Bullinger M Fukuhara S Kaasa S Tests of data quality, scaling assumptions, and reliability of the SF-36 in eleven countries: results from the IQOLA Project. International Quality of Life Assessment J Clin Epidemiol 1998 51 1149 1158 10.1016/S0895-4356(98)00106-1 9817132

[B25] Wagner AK Gandek B Aaronson NK Acquadro C Alonso J Apolone G Bullinger M Bjorner J Fukuhara S Kaasa S Cross-cultural comparisons of the content of SF-36 translations across 10 countries: results from the IQOLA Project. International Quality of Life Assessment J Clin Epidemiol 1998 51 925 932 10.1016/S0895-4356(98)00083-3 9817109

[B26] Bullinger M [Assessment of health related quality of life with the SF-36 Health Survey] Rehabilitation (Stuttg) 1996 35 XVII XXVII quiz XXVII-XXIX 8975342

[B27] Wang HM Beyer M Gensichen J Gerlach FM Health-related quality of life among general practice patients with differing chronic diseases in Germany: cross sectional survey BMC Public Health 2008 8 246 10.1186/1471-2458-8-246 18638419 PMC2515099

[B28] Linacre JM A user's guide to Winsteps/Ministeps Rasch-Model Pograms 2005 Chicago, IL: MESA Press22167686

[B29] Linacre JM Winsteps Rasch measurement computer program Book Winsteps Rasch measurement computer program 2008 (Editor ed.^eds.). City: http://www.winsteps.com/index.htm

[B30] Bond TG Fox CM Applying the Rasch model: Fundamental Measurement in the Human Sciences 2001 London: Lawrence Erlbaum Associates

[B31] Pesudovs K Burr JM Harley C Elliott DB The development, assessment, and selection of questionnaires Optom Vis Sci 2007 84 663 674 10.1097/OPX.0b013e318141fe75 17700331

[B32] Pierce CA Block RA Aguinis H Cautionary Note on Reporting Eta-Squared Values from Multifactor ANOVA Designs Educational and Psychological Measurement 2004 64 916 924 10.1177/0013164404264848

[B33] Linacre JM Sample Size and Item Calibration Stability Rasch Measurement Transactions 1994 7 328

[B34] Sloan J Assessing the minimally clinically significant difference: scientific considerations, challenges and solutions COPD 2005 2 57 62 10.1081/COPD-200053374 17136963

[B35] Norman G Sloan J Wyrwich K Interpreation of changes in health-related quality of life: the remarkable universality of half a standard deviation Med Care 2003 41 582 592 12719681 10.1097/01.MLR.0000062554.74615.4C

[B36] Lamoureux EL Chong E Wang JJ Saw SM Aung T Mitchell P Wong TY Visual impairment, causes of vision loss, and falls: the singapore malay eye study Invest Ophthalmol Vis Sci 2008 49 528 533 10.1167/iovs.07-1036 18234995

[B37] Williams RA Brody BL Thomas RG Kaplan RM Brown SI The psychosocial impact of macular degeneration Arch Ophthalmol 1998 116 514 520 9565052 10.1001/archopht.116.4.514

[B38] Wynia K Middel B van Dijk JP De Keyser JH Reijneveld SA The impact of disabilities on quality of life in people with multiple sclerosis Mult Scler 2008 14 972 980 10.1177/1352458508091260 18632779

[B39] Evenhuis HM Sjoukes L Koot HM Kooijman AC Does visual impairment lead to additional disability in adults with intellectual disabilities? J Intellect Disabil Res 2009 53 19 28 10.1111/j.1365-2788.2008.01114.x 18771511

[B40] Lamoureux EL Tai ES Thumboo J Kawasaki R Saw SM Mitchell P Wong TY Impact of diabetic retinopathy on vision-specific function Ophthalmology 117 757 765 10.1016/j.ophtha.2009.09.035 20122736

[B41] Gall C Mueller I Gudlin J Lindig A Schlueter D Jobke S Franke GH Sabel BA Vision- and health-related quality of life before and after vision restoration training in cerebrally damaged patients Restor Neurol Neurosci 2008 26 341 353 18997310

[B42] Evenhuis HM Medical aspects of ageing in a population with intellectual disability: I. Visual impairment J Intellect Disabil Res 1995 39 Pt 1 19 25 7719057 10.1111/j.1365-2788.1995.tb00909.x

[B43] Finger RP Charbel Issa P Ladewig M Holz FG Scholl HP Intravitreal bevacizumab for choroidal neovascularisation associated with pseudoxanthoma elasticum Br J Ophthalmol 2008 92 483 487 10.1136/bjo.2007.129916 18369065

[B44] Jacobson AM Rand LI Hauser ST Psychologic stress and glycemic control - a comparison of patients with and without proliferative diabetic retinopathy Psychosomatic Medicine 1985 47 372 381 4023165 10.1097/00006842-198507000-00007

[B45] Bernbaum M Albert SG Duckro PN Psychosocial profiles in patients with visual impairment due to diabetic-retinopathy Diabetes Care 1988 11 551 557 10.2337/diacare.11.7.551 3203572

[B46] Lamoureux EL Pallant JF Pesudovs K Rees G Hassell JB Keeffe JE The effectiveness of low-vision rehabilitation on participation in daily living and quality of life Invest Ophthalmol Vis Sci 2007 48 1476 1482 10.1167/iovs.06-0610 17389474

[B47] Hinds A Sinclair A Park J Suttie A Paterson H Macdonald M Impact of an interdisciplinary low vision service on the quality of life of low vision patients Br J Ophthalmol 2003 87 1391 1396 10.1136/bjo.87.11.1391 14609841 PMC1771889

[B48] Reeves BC Harper RA Russell WB Enhanced low vision rehabilitation for people with age related macular degeneration: a randomised controlled trial Br J Ophthalmol 2004 88 1443 1449 10.1136/bjo.2003.037457 15489491 PMC1772403

[B49] Castori M Camerota F Celletti C Grammatico P Padua L Quality of life in the classic and hypermobility types of Ehlers-Danlos syndrome [corrected] Ann Neurol 67 145 146 author reply 146-147 10.1002/ana.21934 20186845

[B50] Swamy BN Chia EM Wang JJ Rochtchina E Mitchell P Correlation between vision- and health-related quality of life scores Acta Ophthalmol 2009 87 335 339 10.1111/j.1755-3768.2008.01203.x 18937810

[B51] Mangione CM Lee PP Pitts J Gutierrez P Berry S Hays RD Psychometric properties of the National Eye Institute Visual Function Questionnaire (NEI-VFQ). NEI-VFQ Field Test Investigators Arch Ophthalmol 1998 116 1496 1504 9823352 10.1001/archopht.116.11.1496

[B52] Bradley C Importance of differentiating health status from quality of life Lancet 2001 357 7 8 10.1016/S0140-6736(00)03562-5 11197385

[B53] Cohen J Statistical power analysis for the behavior sciences 1988 2 London: Taylor & Francis

